# CONNET: Accurate Genome Consensus in Assembling Nanopore Sequencing Data via Deep Learning

**DOI:** 10.1016/j.isci.2020.101128

**Published:** 2020-05-01

**Authors:** Yifan Zhang, Chi-Man Liu, Henry C.M. Leung, Ruibang Luo, Tak-Wah Lam

**Affiliations:** 1 Department of Computer Science, The University of Hong Kong, Hong Kong, China

**Keywords:** Genomics, Bioinformatics, Sequence Analysis

## Abstract

Single-molecule sequencing technologies produce much longer reads compared with next-generation sequencing, greatly improving the contiguity of *de novo* assembly of genomes. However, the relatively high error rates in long reads make it challenging to obtain high-quality assemblies. A computationally intensive consensus step is needed to resolve the discrepancies in the reads. Efficient consensus tools have emerged in the recent past, based on partial-order alignment. In this study, we discovered that the spatial relationship of alignment pileup is crucial to high-quality consensus and developed a deep learning-based consensus tool, CONNET, which outperforms the fastest tools in terms of both accuracy and speed. We tested CONNET using a 90× dataset of *E. coli* and a 37× human dataset. In addition to achieving high-quality consensus results, CONNET is capable of delivering phased diploid genome consensus. Diploid consensus on the above-mentioned human assembly further reduced 12% of the consensus errors made in the haploid results.

## Highlights

• Deep learning methods outperform existing approaches in assembly consensus
• Spatial relationships in alignment pileup are crucial to high-quality consensus
• Diploid consensus can further reduce errors made in haploid consensus
• CONNET can be used for both consensus and polishing

## Graphical Abstract

**Figure:**
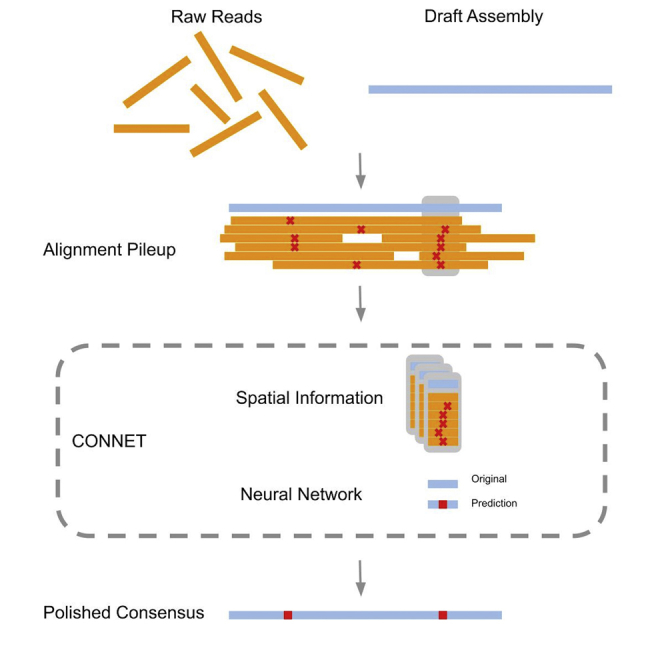

## Introduction

Single-molecule sequencing (SMS) technologies produce much longer reads compared with next-generation sequencing, greatly improving the contiguity of *de novo* assembly of genomes. However, the relatively high error rates in long reads make it challenging to obtain high-quality assemblies. A computational-intensive consensus step is therefore required to resolve the discrepancies in the reads and improve assembly accuracy.

To resolve their discrepancies, reads are usually aligned to the draft assembly. A trivial consensus step could be implemented by taking base-by-base majority votes at each position of the alignment pileup. Such a method is succinct but vulnerable to systematic bias and high indel rates. As the techniques in sequence alignment advance, a partial order alignment graph is found to be useful in improving consensus (Lee et al., 2002). There are several efficient consensus tools (Vaser et al., 2017, Koren et al., 2017, Ruan and Li, 2020) based on this idea, and Racon (Vaser et al., 2017) is generally considered to be the fastest. When paired with a fast assembler miniasm (Li, 2016), miniasm + Racon is the most efficient assembly pipeline. It has also been observed that Racon^(4)^ (i.e., iterate Racon 4 times) often achieves the highest accuracy. Recently, Oxford Nanopore has released a recurrent neural network (RNN)-based consensus tool medaka (ONT, 2018) that is able to further polish the output assembly of Racon^(4)^.

Despite medaka's great success, we still managed to find some room for improvement. Medaka adopts an RNN framework. Alignment pileup is treated as sequential data and converted to input tensors per genomic position. We found that much information is lost during this conversion. We took advantage of the previously overlooked spatial relationship of alignment pileup in our deep learning-based consensus tool, CONNET. We used a sliding window of size 3, instead of size 1, for input tensor construction. Benefitting from more information being captured in the input tensors, our tool can deliver higher quality consensus more efficiently. Compared with medaka's network, our network has one fewer RNN layer, and our RNN cells are half the size.

Furthermore, medaka shows a contingent improvement when polishing assembly results from other tools. Medaka's accuracy was shown to be sensitive to the accuracy of the input assembly in our experiments. In the worst case, it even underperformed Canu. CONNET is designed to achieve an absolute improvement in accuracy. CONNET's network can handle input assemblies ranging from low accuracy to high accuracy, and polish them, giving a consistently high-quality result.

Another challenge in Oxford Nanopore consensus is non-uniform sequencing errors in the reads. The deletion rate (5.74%) is more than twice as high as the insertion rate (2.48%), according to the error profile generated using Nanosim (Yang et al., 2017) on a human genome chr20 (Jain et al., 2018), making recovering missing bases in consensus more challenging. We address this problem by introducing an extra base-recovery phase in our consensus pipeline. In medaka's *E. coli* consensus, deletions (missing bases in assembly) account for more than 75% of total errors. Compared with medaka's results, CONNET's result halved the deletion rate.

Here in this study, we present CONNET, an accurate and flexible consensus tool. CONNET showed the highest accuracy of any existing method and ran faster than the tools with comparable accuracy. For *E. coli*, CONNET improved the accuracy of an *E. coli de novo* assembly from miniasm from 88.65% to 99.92% in 0.52 CPU h, better than Racon^4^+medaka, which achieved 99.85% accuracy in 1.30 CPU h. On a 24 CPU-core machine, CONNET achieved 99.80% accuracy on human chromosome 1 in 1.55 h, whereas Racon^4^ + medaka achieved 99.77% accuracy in 3.27 h. For comparison purposes, our trivial consensus tool achieved 98.68% accuracy on *E. coli* and 98.76% accuracy on human assembly.

Finally, CONNET can generate phased diploid genome consensus for Oxford Nanopore data, further boosting the accuracy of the resulting assembly in diploid organisms such as *Homo sapiens*.

## Results

To demonstrate the high-quality consensus produced by CONNET, we performed experiments on *E. coli* and the human genome. In all the experiments, CONNET was able to achieve the highest accuracy, while being the fastest of the tools with comparable accuracy. We also extended CONNET to a diploid genome consensus tool and benchmarked on several chromosomes of the human genome assembly. The datasets used in the article and their links are summarized in both the “Data and Code Availability” section and Table 5.

### Consensus on *E. coli* Genome

We benchmarked CONNET against other tools, including medaka, Racon, Canu, and wtdbg2, on two Oxford Nanopore *E. coli* datasets. Among the tools benchmarked, Canu and wtdbg2 are both complete genome assemblers that contain a built-in consensus step. CONNET, as well as medaka and Racon, are consensus tools that take draft assembly instead of raw reads as input, and therefore need to be coupled with an assembler in this experiment. Miniasm (Li, 2016) exactly suits this purpose, as it is a long read *de novo* assembler that does not contain a consensus step. Moreover, miniasm + Racon pipeline is the recommended usage of Racon. For medaka, the default usage is pomoxis + medaka, where pomoxis is a wrapper of miniasm + Racon.

**Figure 1:**
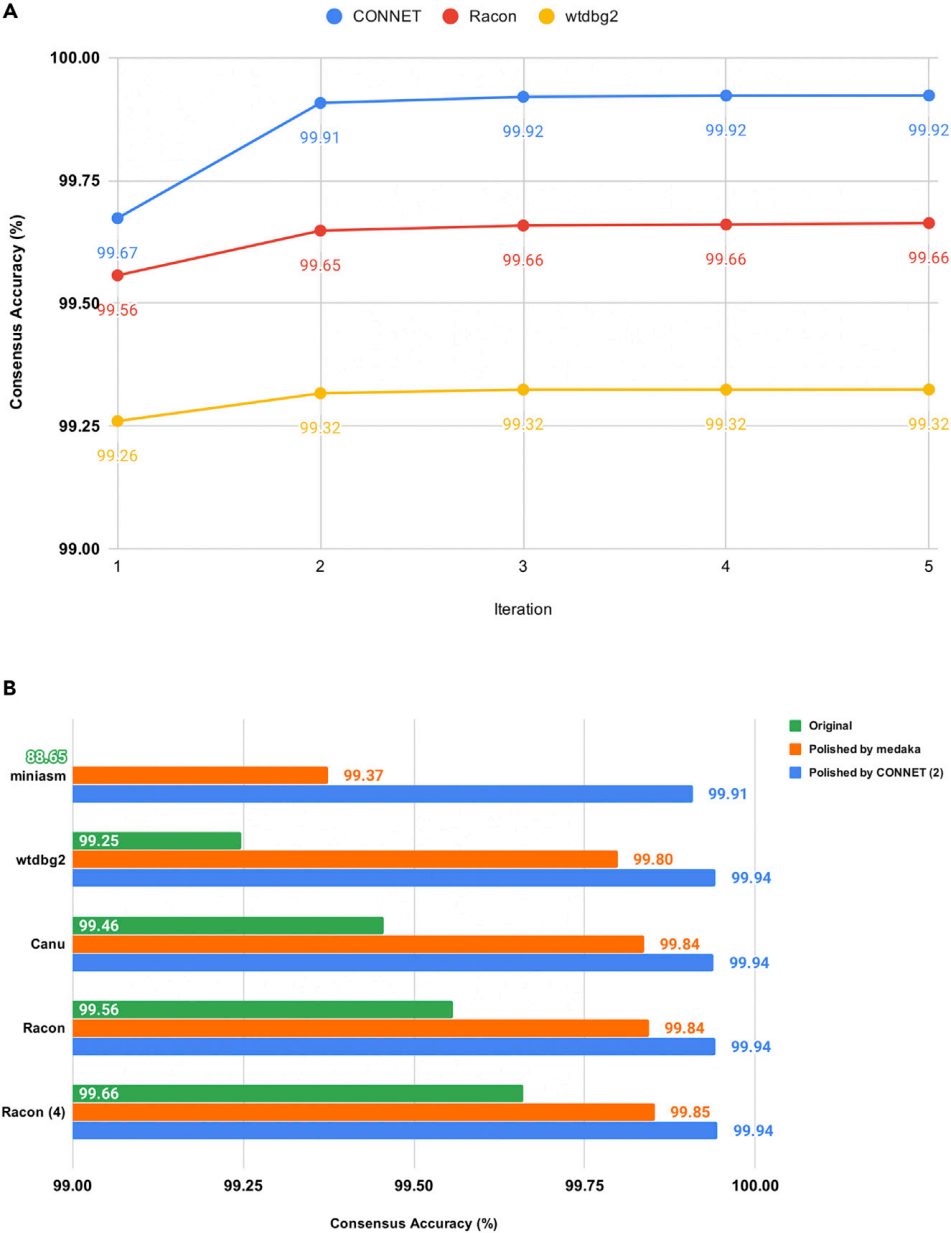
Consensus Accuracy on the 90× *E. coli* SCS110 R9.4.1 Dataset (A and B) CONNET and Racon worked on draft assembly produced by miniasm. (A) CONNET has achieved a higher accuracy by using fewer iterations than other iterateable consensus tools. (B) CONNET has further improved the accuracy to over 99.90% after polishing the consensus generated by various other tools.

We obtained a publicly available 90× *E. coli* SCS110 dataset generated with the R9.4.1 sequencing chemistry. Table 1 summarizes the assembly and consensus results. Identities were evaluated against the *E. coli* SCS110 reference genome using QUAST (Gurevich et al., 2013). A number in the superscript parenthesis after an iterable tool indicates how many iterations the tool has been run. CONNET was trained on the same dataset due to the scarcity of publicly available R9.4.1 *E. coli* datasets. In Table 2 and Figure 3, we show results using different datasets or chromosomes for training and testing. However, Figure 1B has shown that CONNET has learnt a general model for *E. coli* assembly, and an agnostic of the algorithm used for generating its input. CONNET was trained on the draft assembly generated by miniasm and performed equally well, using wtdbg2, racon, and Canu as its input.

**Table 1:** The Consensus Results of the 90× *E. coli* SCS110 R9.4.1 Dataset

Pipeline	# Contigs	Total Bases (bp)	Identity (%)	Time (min)
miniasm + CONNET^(2)^	1	4,710,898	99.908	31
miniasm + Racon^(4)^ +medaka	1	4,711,032	99.852	78
miniasm + Racon^(4)^	1	4,702,069	99.661	65
Canu	8	4,813,634	99.444	707
wtdbg2^(2)^	1	4,684,197	99.325	22

**Table 2:** The Consensus Results of Three Subsampled *E. coli* K-12 R9 Datasets

Dataset	Pipeline	# Contigs	Total Bases (bp)	Identity (%)	Time (min)
Training set (54×)	miniasm + CONNET^(2)^	1	4,619,544	99.817	26
Training set (54×)	miniasm + Racon^(4)^	1	4,636,480	99.498	42
Training set (54×)	wtdbg2^(2)^	1	4,612,292	99.403	23
Training set (54×)	Canu	6	4,690,706	99.523	541
Testing set #1 (60×)	miniasm + CONNET^(2)^	1	4,625,887	99.814	31
Testing set #1 (60×)	miniasm + Racon^(4)^	1	4,627,221	99.617	48
Testing set #1 (60×)	wtdbg2^(2)^	1	4,613,955	99.487	25
Testing set #1 (60×)	Canu	3	4,645,786	99.586	490
Testing set #2 (60×)	miniasm + CONNET^(2)^	3	4,657,298	99.797	30
Testing set #2 (60×)	miniasm + Racon^(4)^	3	4,663,034	99.593	46
Testing set #2 (60×)	wtdbg2^(2)^	1	4,612,282	99.473	24
Testing set #2 (60×)	Canu	3	4,659,585	99.577	469

CONNET achieved the highest accuracy, 99.92%, followed by medaka's 99.85% accuracy. Figure 1A compared the performance of iterable tools: CONNET, wtdbg2, and Racon. The performance of all tools was increasing and converging. However, other tools could not achieve the accuracy of CONNET after a sufficient number of iterations.

**Figure 2:**
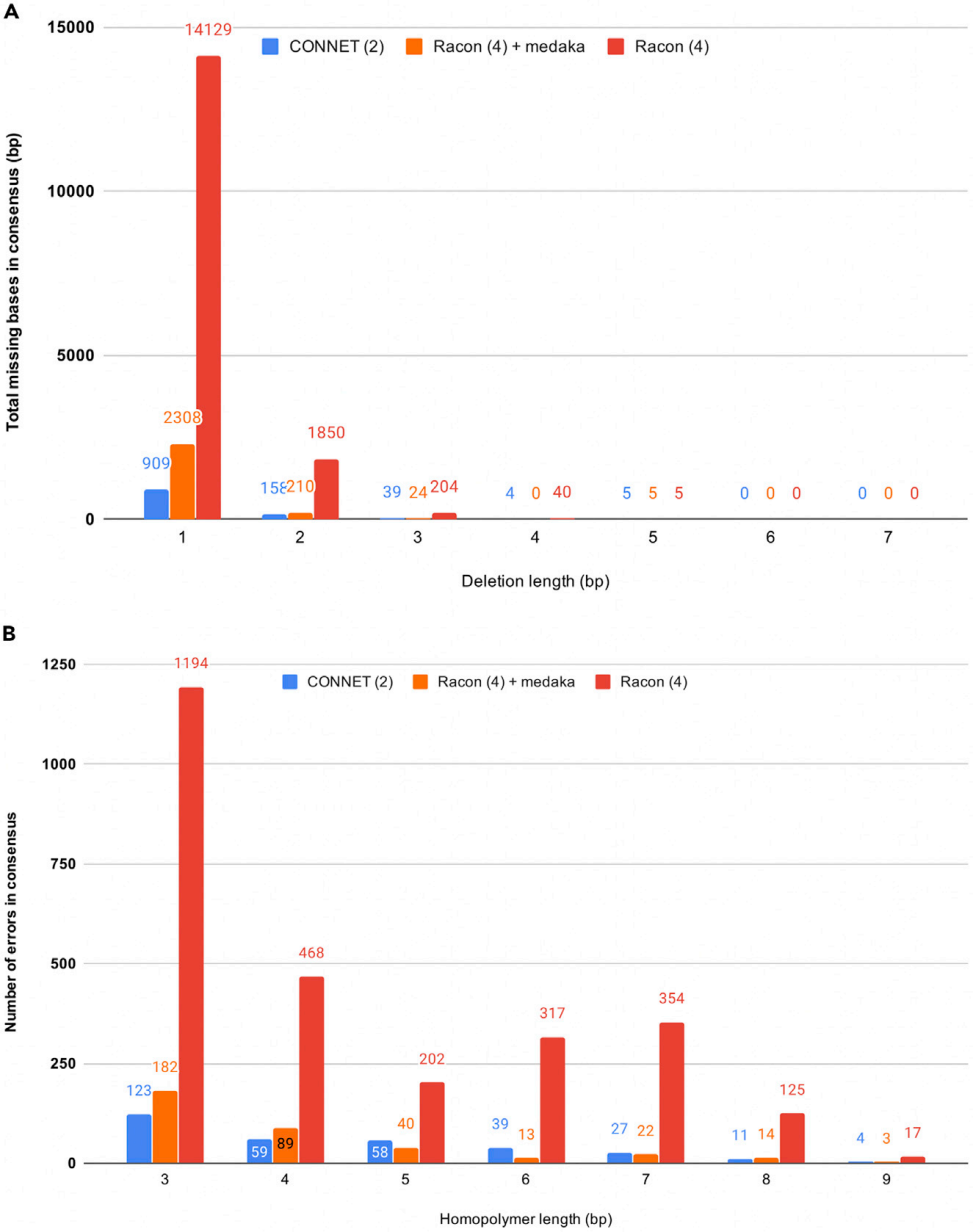
Consensus Error Analysis Using the 90× *E. coli* SCS110 R9.4.1 Dataset (A) Missing bases (deletions) in consensus, grouped by deletion length. (B) Homopolymer errors in consensus, grouped by homopolymer length.

**Figure 3:**
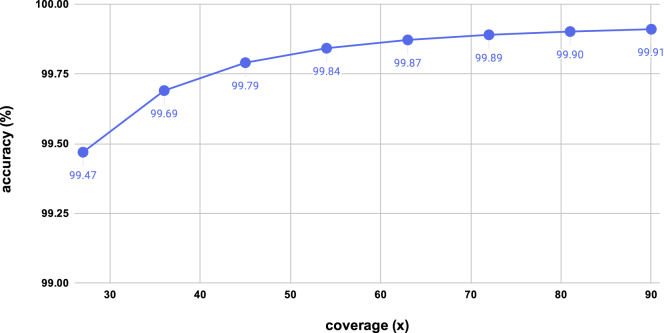
Consensus Accuracy at Different Coverages on *E. coli* SCS110 Using CONNET We used a CONNET model trained at 90×.

We analyzed the errors in consensus with Figures 2A and 2B. Owing to its unique base-recovery phase, CONNET was the best tool for reducing deletion errors. CONNET and medaka were the most effective tools for resolving homopolymer errors. Medaka has proposed a specialized “homopolymer compression” algorithm, whereas CONNET's strength with homopolymer accuracy comes from our novel spatial-aware input tensor. We further studied the impact of sequencing coverage on consensus accuracy in Figure 3. We downsampled the dataset used in Table 1 to different coverages ranging from 27× to 81×. As expected, consensus accuracy falls as coverage decreases. However, at 63×, or 70% of coverage of the original dataset, CONNET achieves a higher accuracy than other tools on the original 90× dataset. At 36×, or as low as 40% of the original coverage, CONNET still outperforms partial order alignment-based tools at 90v. CONNET enables accurate consensus at a lower coverage, resulting in lower sequencing costs.

As deep learning-based tools were shown to be more accurate, we further polished the results of all tools using CONNET and medaka, in Figure 1B. The performance of CONNET was stable and consistent. After two iterations, CONNET polished all results to over 99.90% accuracy. On the other hand, when a noisy input assembly (of 88.65% accuracy) was provided, medaka was not able to compute a high-quality consensus accurately, as it underperformed on graph-based methods, achieving only 99.37% accuracy. Despite the fact that all accuracy improved after being polished by medaka, the consensus accuracy of medaka was largely determined by the accuracy of input draft assembly. When given its own result as input, medaka failed to improve the accuracy further, whereas CONNET was able to continue to improve medaka's accuracy. We also observed that CONNET can benefit from pairing with other consensus tools, as its performance has improved to 99.94%, whereas the performance of medaka was capped at 99.85%.

To demonstrate that CONNET was not overfitted to its training data, we also benchmarked another publicly available *E. coli* K-12 dataset released in 2015. The dataset is based on an earlier (and obsolete) R9 sequencing chemistry, and the same dataset has been used in previous studies (Loman et al., 2015, Vaser et al., 2017). We subsampled the dataset into three datasets with similar coverage (54×, 60×, 60×, respectively) for performance cross-validation. Using the CONNET model trained with one of the subsampled dataset (54×), our results showed that CONNET outperformed all other tools. Table 2 summarized the performance of different tools. Medaka was excluded, as it does not provide a model for R9 data. CONNET achieved the highest accuracy of 99.80% of all tools for all three datasets.

### Consensus on the Human Genome

To extend our discussion to larger and more complicated genomes, we benchmarked CONNET, Racon, and medaka on data from the Whole Human Genome Sequencing Project (Jain et al., 2018). Our reference genome for evaluation was obtained by applying the known variants of sample NA12878 (Zook et al., 2014) to the human reference genome GRCh37.

**Figure 4:**
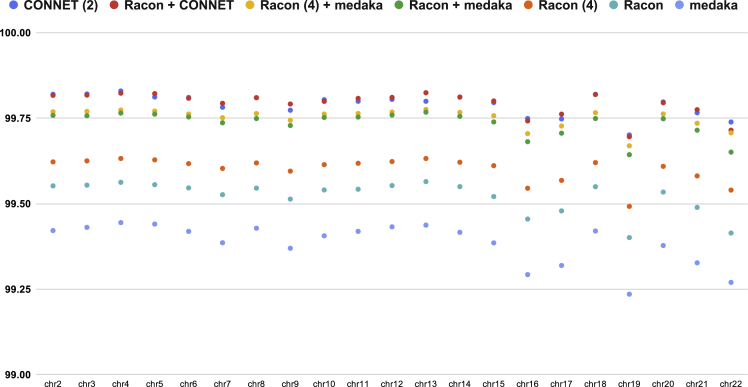
Consensus Accuracy on Human Chromosome 2 to 22 (Using a CONNET Model Trained on Human Chromosome 1) All consensus tools worked on the same draft assembly produced by miniasm.

Table 3 summarizes the performance of different tools on chromosome 1. All tools were benchmarked on a 24-core Intel(R) Xeon(R) Silver 4116 CPU @ 2.10GHz workstation. Assembly time was excluded from the running time, whereas all pipelines were started from the same miniasm draft assembly. As shown in Table 1, medaka spent most of its time on four iterations of Racon. It might be asked whether the running time could be optimized by reducing the number of Racon iterations while keeping a comparable performance. We therefore benchmarked three different versions of medaka pipelines, with the number of Racon iterations being 4 (default), 1, and 0, respectively. Other than miniasm + CONNET, we also introduced a miniasm + Racon + CONNET pipeline in the benchmark as CONNET can benefit from being paired with other consensus tools. The model of CONNET was trained on chromosome 1, and Figure 4 shows the accuracy of all other autosomes (chromosome 2 to 22). As shown, CONNET consistently outperformed the others, achieving over 99.80% accuracy on average.

**Table 3:** The Consensus Results of Human Chromosome 1

Consensus Tool	# Contigs	Total Bases (bp)	Identity (%)	Time (min)
CONNET^(3)^	31	222,090,834	99.811	146
CONNET^(2)^	31	222,072,948	99.804	93
Racon + CONNET	32	223,108,173	99.794	77
Racon^(4)^ + medaka	34	223,337,382	99.756	196
Racon + medaka	33	223,316,142	99.745	114
CONNET	31	222,163,331	99.708	48
Racon^(4)^	32	223,030,646	99.610	111
Racon	32	222,872,015	99.536	29
Medaka	33	223,659,895	99.411	85

All consensus tools worked on the same draft assembly produced by miniasm.

To test the generality of the model that CONNET has learned, we performed a cross-species benchmark by applying the model learned from human chromosome 1 (the same as the one used in Table 3) on the *E. coli* genome consensus. Table 4 shows that the model learnt from the human genome performs well on the simpler *E. coli* genome.

**Table 4:** The Consensus Results of the 90× E. coli SCS110 R9.4.1 Dataset Using a CONNET Model Trained from Human Chromosome 1

Pipeline	# Of Contig	Total Bases (bp)	Identity (%)
miniasm + CONNET^(1)^	1	4,707,712	99.723
miniasm + CONNET^(2)^	1	4,707,035	99.894

### Diploid Consensus on the Human Genome

We noticed that the performance of all tools dropped in the human genome assembly, compared with the *E. coli* assembly. One possible reason is the diploidy of the human genome. Existing consensus tools may be confused in diploid regions if they are programmed to output one best consensus sequence. Based on our haploid genome consensus component, we extended CONNET to a phased diploid genome consensus tool with the help of a read-based variant phasing tool, Whatshap (Martin et al., 2016). We have proposed a new measurement of accuracy (in Methods) for diploid genome assembly.

To determine the extent to which a diploid consensus is better than its haploid counterpart, we used the following statistics. We defined $L_{h}$ as the aligned length and $A_{h}$ as the accuracy of a haploid consensus. For a diploid consensus, two haploids will be generated. Thus, we defined its aligned length $L_{d}$ as the arithmetic mean of the aligned length of the two haploids. Its accuracy $A_{d}$ was calculated as described in Methods. We assumed all bases are independent, so that the comparison of $A_{h}$ and $A_{d}$ can be viewed as a Two Proportion z-test. The pooled proportional was calculated as $\frac{L_{h}\;A_{h}+L_{d}\;A_{d}\;}{L_{h}+L_{d}\;}$. The lower the p value, the more significant the performance of the diploid consensus deviates from the haploid.

**Figure 5:**
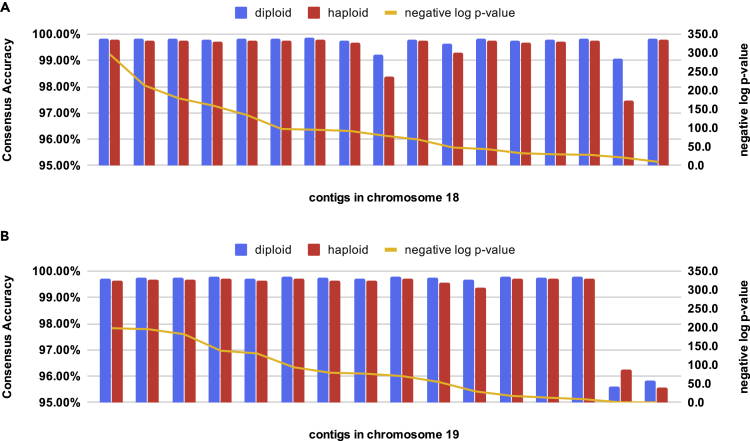
Comparison of the Haploid and Diploid Consensuses Produced by CONNET (A) Contigs in chromosom 18. (B) Contigs in chromosome 19. In draft assembly, chromosomes are fragmented into several contigs of varying lengths. Each pair of blue and red bars represents one same contig. The contigs are sorted in the ascending order of p value, which indicates how significant the accuracy difference is between the haploid and the diploid versions.

Figure 5 showed the accuracy of each contig when diploid genome consensus was applied to human chromosomes 18 and 19. As shown, diploid contigs are more accurate than the corresponding haploid contigs in all but one contig (the second shortest contig in chromosome 19, where the p value is 0.40: diploid consensus got 7 incorrect bases out of 159 bp, whereas haploid consensus got 6 out of 160 bp). In other contigs, the p value can be as low as 1e-300 (the first contig of chromosome 18 has a length of 15.1 Mbp, diploid consensus got 24.9 kbp incorrect bases and haploid got 33.8 kbp), indicating that diploid consensus is significantly more accurate than haploid consensus. On the two chromosomes we have tested, diploid consensus further reduced the total errors in haploid consensus by about 12%.

## Discussion

We have described CONNET, a deep-learning based diploid consensus tool. We have shown that CONNET is more accurate than other state-of-the-art consensus tools and is capable of achieving an absolute improvement in accuracy that does not depend on the quality of input draft assembly.

We demonstrate that deep neural network methods have the potential to better solve the consensus problem. Compared with partial order alignment-based methods, deep neural networks are more flexible as they do not rely on heuristic rules to determine the most probable sequence. Deep neural networks are also less vulnerable to systematic bias in sequencing, as it is feasible to train multiple models for various sequencing methods.

CONNET is the first deep-learning based method to make use of spatial relationships in alignment pileup. A sliding window of 3 instead of size 1 is used for input tensor construction. Compared with the other deep-learning based method, medaka, CONNET has one fewer layer of BRNN and manages to achieve better accuracy. Our input tensor efficiently captures interesting features in alignment pileup, which makes the neural network easier to learn. Furthermore, our experiments show that CONNET has learnt a generalized model except for sequencing chemistry. CONNET is agnostic of the algorithm used for generating its input, and the model trained on the human genome is applicable to the simpler *E. coli* genome.

CONNET has achieved the highest consensus accuracy, and it is able to further polish existing results from other assembly or consensus tools. A more accurate genome assembly would be useful for other bioinformatics problems such as variant calling. We have also studied the role that sequencing coverage played in consensus accuracy. As a consequence, a lower coverage is sufficient for CONNET to maintain the same level of accuracy as other state-of-the-art tools, reducing the cost involved in sequencing.

Currently, CONNET is designed for Nanopore sequencing data. In the future, we may extend its application to sequencing data from other platforms such as PacBio.

### Limitations of the Study

We designed CONNET to deliver better performance in consensus accuracy. We intend in our future work to focus not only on accuracy but also on the improvement of genome assembly, including better continuity.

In our implementation, we chose a sliding window of size 3 for input tensor construction. Ideally, a larger sliding window that might be able to capture more spatial relationships is preferred. However, considering the memory limit of a typical Graphics Processing Unit used for training, better encoding of the input tensor is required to enable a larger sliding window.

### Resource Availability

#### Lead Contact

Further information and requests for resources should be directed to and will be fulfilled by the Lead Contact, Ruibang Luo (rbluo@cs.hku.hk).

#### Materials Availability

This study did not generate new unique reagents.

#### Data and Code Availability

CONNET is made freely available to the research community at https://github.com/HKU-BAL/CONNET.

The datasets used in the article and their links are summarized in Table 5.

**Table 5:** Summary of the Datasets Used in Our Study

Dataset	Sequencing	Coverage	URL
*E. coli* SCS110	R9.4.1	90×	https://s3-eu-west-1.amazonaws.com/ont-research/medaka_walkthrough_no_reads.tar.gz
*E. coli* K-12	R9	174×	http://lab.loman.net/2015/09/24/first-sqk-map-006-experiment/
*H. Sapiens*	R9.4.1	37×	https://github.com/nanopore-wgs-consortium/NA12878/blob/master/Genome.md

## Methods

All methods can be found in the accompanying Transparent Methods supplemental file.

## Supplemental Information

Document S1. Transparent Methods and Figures S1–S4
