# Supplementary material for: CONNET: Accurate Genome Consensus in Assembling Nanopore Sequencing Data via Deep Learning
Source: iScience. 2020 May 1;23(5):101128. doi: 10.1016/j.isci.2020.101128 (PMC7229283; doi:10.1016/j.isci.2020.101128)
Supplement: Document S1. Transparent Methods and Figures S1–S4 [file mmc1.pdf]

iScience, Volume *23*

Supplemental Information

CONNET: Accurate Genome Consensus in Assembling Nanopore Sequencing Data via Deep Learning

Yifan Zhang, Chi-Man Liu, Henry C.M. Leung, Ruibang Luo, and Tak-Wah Lam

Supplementary Figures

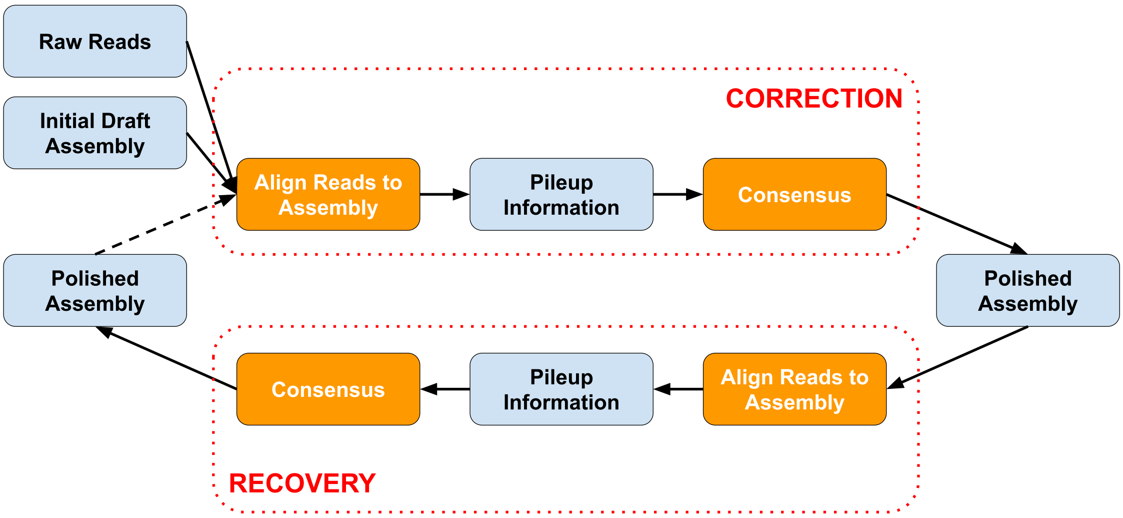

Supplementary Figure 1. CONNET's workflow. Related to "Transparent Methods – Workflow".

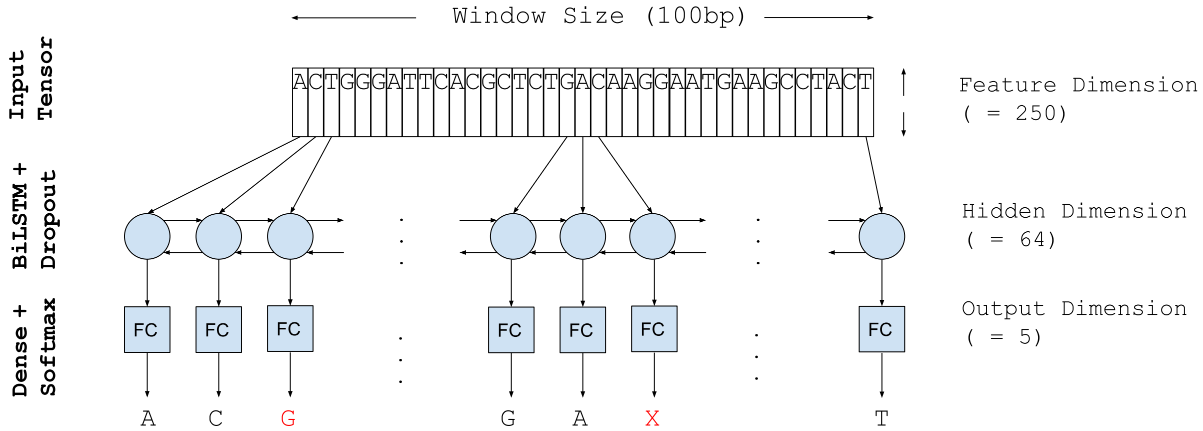

Supplementary Figure 2. The neural network architecture of the correction phase. Related to "Transparent Methods – Neural network architecture".

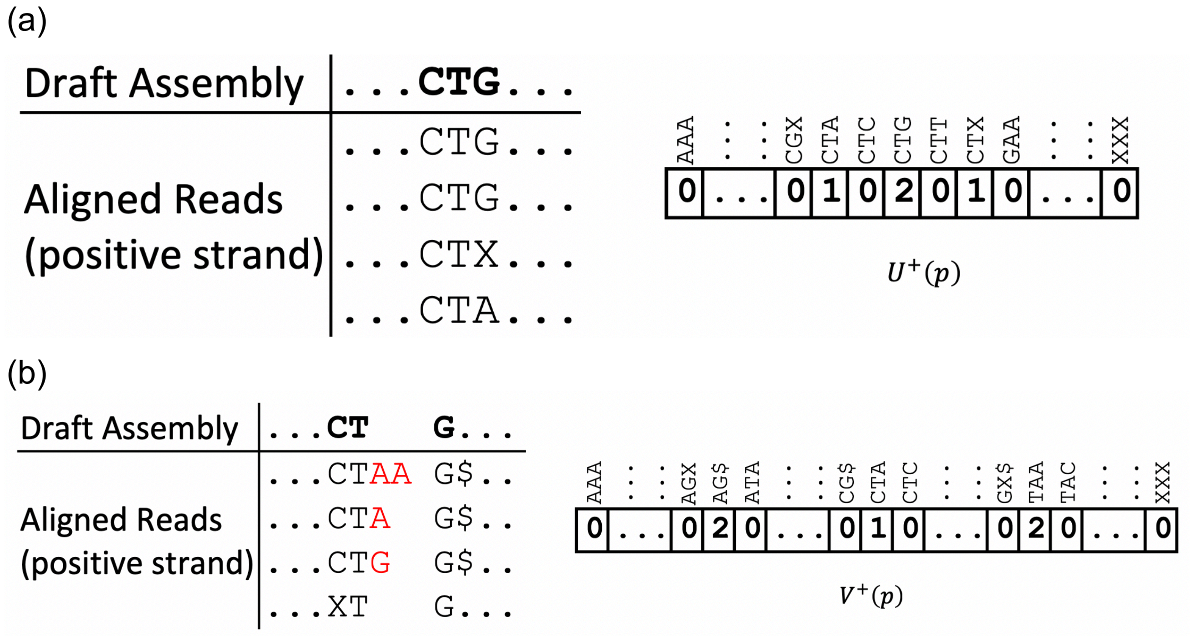

Supplementary Figure 3. Illustration of the input tensors. (a) An example of alignment pileup and its corresponding input tensor at the correction phase. (b) An example of alignment pileup and its corresponding input tensor (partial) at the recovery phase. Spaces were added for better visualization. Insertions are in red. Related to “Transparent Methods – Spatial relationship of alignment pileup”.

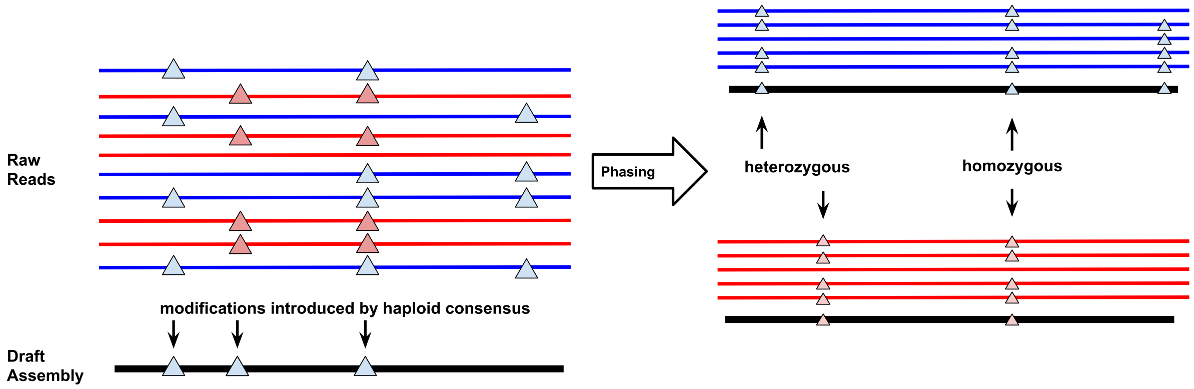

Supplementary Figure 4. A schematic illustration of the diploid consensus module. The left part of the figure shows the haploid consensus workflow. The “phasing” is achieved by applying WhatsHap to the raw reads and the haploid consensuses at the positions where variants were found. The right part of the figure shows that raw reads are partitioned into two groups by WhatsHap. Consensuses will then be computed from the two groups of reads, respectively. Related to “Transparent Methods – Diploid consensus module”.

Transparent Methods

Workflow

There are three types of consensus errors: mismatch (incorrect nucleotide), insertion (extra nucleotide), and deletion (missing nucleotide). We discovered that errors from existing tools are mainly in the form of deletions. Therefore, we designed a pipeline to reduce deletions in our consensus. We separated our consensus module into two phases. The first phase, *correction*, aims at correcting the mismatching bases in the assembly and removing extra bases; the second phase, *recovery*, aims at recovering the bases that are lost during assembly construction. In the *recovery* phase, we also invented a method to capture spatial relationships in inconsistent regions of the alignment pileup, where there are many discrepancies in the reads.

Workflow is described in Supplementary Figure 1. CONNET is an iterative consensus tool. It takes raw reads and a draft assembly as input, and outputs a polished assembly. Each iteration consists of two phases, *correction* and *recovery*, while each phase consists of one alignment step and one consensus step. In both alignment steps, raw reads are aligned to current draft assembly sequences to provide pileup information needed in the subsequent consensus step. Both consensus steps make use of neural networks. The networks take as input the encoded spatial relationship in alignment pileup. *Correction* network predicts the nucleotide (A, C, G, T, or X, which denotes a deletion) at each genomic position of the current draft assembly. *Recovery* network predicts the number of missing bases at each position. Lost bases are then recovered using pileup information.

Neural network architecture

CONNET adopts a bidirectional recurrent neural network (BRNN) (Schuster and Paliwal, 1997) architecture for alignment pileup. As assembly sequences come in variable lengths, we have chosen recurrent neural network as a starting point. Similar to a time series, genomic sequences have a direction as well. Due to the possible strand bias in sequencing technology, we may expect the characteristics of forward strands and reverse strands to be nonidentical. BRNN, which processes the input data in both positive and negative direction, is used to capture strand-specific information.

Our neural network architecture for the *correction* and the *recovery* phase is identical except for parameters like output dimension. Supplementary Figure 2 shows settings used in the *correction* phase. Each genomic position in the draft assembly corresponds to one neuron in the input layer and then corresponds to one node in the BRNN layer. We choose bidirectional long short-term memory (LSTM) (Hochreiter and Schmidhuber, 1997) as the BRNN layer for its capability of remembering long sequences. The output layer of either phase is designed as one neuron per genomic position. As each node in LSTM corresponds to one output neuron, to make our architecture concise, we directly connect them using a single layer. We chose to use a fully connected layer that captures all information from the

previous layer. In order to prevent over-fitting, we inserted a dropout layer after each LSTM node, randomly ignoring some of the hidden units during training.

In the *correction* phase, output tensor represents the probability distribution of the correct nucleotide, A/C/G/T/X, at each genomic position. In the *recovery* phase, output tensor represents the probability distribution of the number of lost bases, $0/1/2/3/4/\geq 5$, at each genomic position.

Both phases perform classification tasks. In the testing process, the class label with highest probability is predicted. In the training process, categorical cross-entropy is used as a loss function. Ground truth is obtained by aligning the reference genome to draft assembly. For each genomic position in the draft assembly, the ground truth of *correction* phase is the aligned nucleotide (or "X" in case of deletion) in reference genome; ground truth of *recovery* phase is set to 0, except at the places where the reference genome is inserted.)

Spatial relationship of alignment pileup

Existing machine learning-based methods (Luo et al., 2019; Luo et al., 2020; Poplin et al., 2018) typically consider columns of alignment pileup independently when constructing the input tensor. The number of occurrences of different nucleotides or probability distribution of nucleotides at a single column is a common approach. This results in a small tensor, and thus little information is encoded in each genomic position. Such representation loses all spatial relationships in the pileup as k-mers cannot be reconstructed from nucleotide counts.

For example, if we know, there are 4 A's and 3 C's at a genome position in the pileup, and 4 G's and 3 T's at the next position. One scenario is there are 4 reads with "AG", and 3 reads with "CT", which may indicate two read groups coming from different haplotypes of a diploid genome. Another scenario would be 2 reads with "AT", 2 reads with "AG", 2 reads with "CG" and another 1 read with "CT", which may indicate misalignment or a high sequencing error rate in the case of a diploid genome. If we use a simple counting method to represent the alignment pileup, our network cannot differentiate between these two scenarios.

To our knowledge, we are the first to use a deep learning-based method to consider the spatial relationship in alignment pileup. We used a sliding window of 3 instead of size 1 for input tensor construction.

Let A be the alignment of query reads Q to reference sequence R. In our case, Q is the raw reads and R is the current draft assembly sequence. For each genomic position $r \in R$, let R_r be the nucleotide in reference sequence and A_r be the alignment pileup at r. We encode information in A_{r-1}, A_r, A_{r+1}, instead of only A_r, when constructing the input neuron I_r for each genomic position r. In our design, the network is expected to output predictions O_r corresponding to I_r.

The **correction** phase

Input neuron I_r consists of two 125-dimensional vectors, U_r^+ and U_r^-, for each genomic position r. Each one of the 125 values in U_r^+ (resp. U_r^-) corresponds to the count of a particular 3-mer centred at r in the alignment pileup, considering only reads aligned to the forward (resp. reverse) strand (Supplementary Figure 3a). In this way, we manage to encode the spatial information stored in A_{r-1}, A_r, A_{r+1} in our input neuron I_r. Here our alphabet consists of five letters: $\Sigma = \{\mathrm{A}, \mathrm{C}, \mathrm{G}, \mathrm{T}, \mathrm{X}\}$, where the first four correspond to nucleotide and the last one "X" represents a gap in alignment pileup. Therefore, we have a total of $|\Sigma|^3 = 125$ possible 3-mers. Each value in U_r^+ (resp. U_r^-) would correspond to such a 3-mer.

The **recovery** phase

In additional to U_r^+ and U_r^- defined in the *correction* phase, input neuron I_r includes two more 150-dimensional vectors, V_r^+ and V_r^-, for each genomic position r. In the same manner as above, the superscript "+" indicates information from the forward strand reads, and "-" indicates information from the reverse strand reads. Each value in , V_r^+ (resp. V_r^-,) corresponds to the count of a particular 3-mer, which overlaps with an insertion in the pileup at r, considering only reads aligned to the forward (resp. reverse) strand (Supplementary Figure 3b). This explains $|\Sigma|^3 = 125$ out of the 150 values in either vector. The remaining 25 values come from another rule: if there is an insertion in the pileup, a special symbol "$" is appended to 3-mer centered at r. By our design, the number of total 3-mers ended in "$" equals to the number of total reads that contains an insertion in the pileup. Intuitively, the 3-mers ended in "$" help us distinguish the scenarios of many reads with short insertions and few reads with long insertions.

Diploid consensus module

CONNET relies on phasing information to generate a diploid genome consensus (Supplementary Figure 4). We start from the haploid consensus result from CONNET. We treat the initial draft assembly as a "reference genome" and treat the modifications our haploid consensus introduced to the draft assembly as "variants". In this setting, we have used WhatsHap for phasing the "variants" in order to separate the raw reads into two groups corresponding to two haplotypes. A diploid consensus can be obtained by applying a haploid consensus to each group.

Implementation of trivial consensus

For each column of pileup: $\mathrm{output} = \mathrm{argmax}_{x \in \Sigma}\{\mathrm{pileup.count(x)}\}$ where $\Sigma = \{\mathrm{A}, \mathrm{C}, \mathrm{G}, \mathrm{T}, \mathrm{X}\}$ and "X" denotes deletion. In case the insertion AF exceeds 20%, the insertion pattern with the highest frequency is inserted to consensus.

Accuracy of diploid genome assembly consensus

We can formulate the problem of diploid genome assembly consensus as detailed below: Given raw reads, draft assembly, and alignment of raw reads to draft assembly, for each contig in draft assembly, output a pair of contigs with preferably higher accuracy representing two sets of chromosomes.

Accuracy for each paired contig $c_{1,2}$ is defined as

$$Accuracy := \max\left(\frac{IDY(r_1, c_1) + IDY(r_2, c_2)}{2}, \frac{IDY(r_1, c_2) + IDY(r_2, c_1)}{2}\right),$$

where $r_{1,2}$ represents two sets of chromosomes in the true genome, and $IDY(\cdot,\cdot)$ is identity measured by QUAST.

Since the length difference of r_1 and $r_2$is negligible compared with the length of r_1 or r_2, we simply take the arithmetic mean instead of the weighted average, with respect to contig length for $IDY(r_1, c_1) + IDY(r_2, c_2)$.

Supplementary References

Hochreiter, S., and Schmidhuber, J. (1997). Long short-term memory. Neural computation *9*, 1735-1780.

Luo, R., Sedlazeck, F.J., Lam, T.-W., and Schatz, M.C. (2019). A multi-task convolutional deep neural network for variant calling in single molecule sequencing. Nature communications *10*, 1-11.

Luo, R., Wong, C.-L., Wong, Y.-S., Tang, C.-I., Liu, C.-M., Leung, C.-M., and Lam, T.-W. (2020). Exploring the limit of using a deep neural network on pileup data for germline variant calling. Nature Machine Intelligence, 1-8.

Poplin, R., Chang, P.-C., Alexander, D., Schwartz, S., Colthurst, T., Ku, A., Newburger, D., Dijamco, J., Nguyen, N., and Afshar, P.T. (2018). A universal SNP and small-indel variant caller using deep neural networks. Nature biotechnology *36*, 983-987.

Schuster, M., and Paliwal, K.K. (1997). Bidirectional recurrent neural networks. IEEE transactions on Signal Processing *45*, 2673-2681.
